# Circulating Tumor DNA-Based Assessment of Minimal Residual Disease in Colorectal Cancer: Prognostic and Predictive Implications

**DOI:** 10.3390/cancers18050754

**Published:** 2026-02-26

**Authors:** Ahmet Anil Ozluk, Will Colley, Zeynep Beyza Arik, Osman Kostek, Aakash Sunkari, Midhun Malla, Mehmet Akce

**Affiliations:** 1 Division of Hematology and Oncology, University of Alabama at Birmingham, Birmingham, AL 35233, USA; ahmetanilozluk@uabmc.edu (A.A.O.); midhunmalla@uabmc.edu (M.M.); 2 Heersink School of Medicine, University of Alabama at Birmingham, Birmingham, AL 35233, USA; wccolley@uab.edu; 3 Department of Dermatology, University of Pittsburgh Medical Center, Pittsburgh, PA 15213, USA; beyzaarik@gmail.com; 4 Department of Medical Oncology, Marmara University School of Medicine, Istanbul 34854, Turkey; osman.kostek@marmara.edu.tr; 5 Department of Neurobiology, University of Alabama at Birmingham, Birmingham, AL 35233, USA; asunkari@uab.edu

**Keywords:** colorectal cancer, circulating tumor DNA, minimal residual disease, prognostic biomarker, predictive biomarker, precision oncology

## Abstract

Circulating tumor DNA (ctDNA) has emerged as a promising and versatile biomarker in colorectal cancer (CRC), providing real-time insights into the tumor burden, minimal residual disease (MRD), and treatment response across both early and metastatic stages. In patients with resected stage II–III CRC, post-operative ctDNA positivity is a robust predictor of recurrence and may outperform traditional clinicopathologic risk factors. It can facilitate adjuvant therapy discussions; however, treatment escalation or de-escalation based solely on ctDNA results is not yet supported by available interventional data. In the metastatic setting, ctDNA-based techniques could provide non-invasive molecular profiling and a monitoring response to systemic therapies. Peripheral blood-based techniques could also help detect emerging resistance to systemic therapy. Emerging evidence highlights that quantitative assessment of ctDNA dynamics, including the baseline burden and post-treatment clearance, could further refine risk stratification and inform treatment personalization. Collectively, ctDNA represents a promising and evolving biomarker with well-established prognostic and emerging predictive potential and is poised to support precision oncology across the continuum of CRC.

## Simple Summary

Circulating tumor DNA (ctDNA) is emerging as a promising blood-based biomarker capable of detecting minimal residual disease in patients with colorectal cancer. The presence or absence of ctDNA following surgery or systemic therapy provides valuable information regarding the risk of disease recurrence. In this review, we summarize the current evidence supporting the prognostic and predictive roles of ctDNA across different stages of colorectal cancer and evaluate key completed studies as well as ongoing randomized trials. We also highlight current limitations and future directions for the integration of ctDNA-guided strategies into routine clinical practice.

## 1. Introduction

Colorectal cancer (CRC) is the third most common cancer in the United States and the third-leading cause of cancer-related deaths in men and the fourth in women. Despite substantial advancements in treatment options, CRC continues to rank among the leading causes of cancer-related mortality [1]. The tumor (T), node (N), and metastasis (M) TNM staging system remains the most significant indicator for determining the stage of the disease and estimating the five-year survival rate. However, traditional prognostic factors (e.g., TNM staging, lymphovascular invasion, tumor grade, and genetic mismatch repair status) provide only indirect and incomplete information about the minimal residual disease (MRD) and future recurrence risk. The five-year survival rate is substantially higher in early-stage CRC than in metastatic disease (91% vs. 14%) [2]. Currently, despite adjuvant chemotherapy, recurrence occurs in 15–30% of patients with stage III and stage II CRC [3]. In metastatic disease, chemotherapeutic and immunotherapeutic approaches based on the tumor molecular profile are the standard treatment [4].

**Figure 1:**
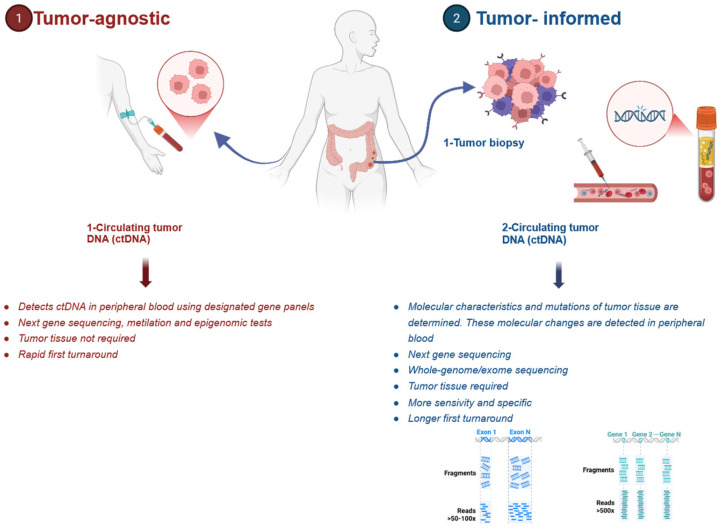
Circulating tumor DNA assay and detection. Illustration created with BioRender.com.

Circulating tumor DNA (ctDNA) represents the tumor-derived fraction of circulating cell-free DNA (cfDNA) detected in peripheral blood and serves as a molecular surrogate of the tumor genome by capturing tumor-specific genomic and epigenomic alterations, including somatic point mutations, copy number alterations, DNA methylation patterns, and structural rearrangements (Figure 1) [5,6]. ctDNA provides another approach to investigate the presence of MRD in the stage II/III CRC patient populations, which serves to evaluate whether there is a benefit of adjuvant chemotherapy. For the assessment of MRD, ctDNA should be evaluated at least 2–4 weeks after curative-intent surgical resection. Long-term ctDNA surveillance is typically performed at 8–12-week intervals [3]. The presence of ctDNA following surgery (positive MRD) is associated with worse recurrence-free survival (RFS). In contrast, the absence of ctDNA (negative MRD) is associated with a lower risk of recurrence [7,8]. Although ctDNA has not yet been established as a routinely validated predictive biomarker in clinical practice, it is actively being investigated for several applications. These include MRD detection, diagnostic support, treatment monitoring, prognostic assessment, and early identification of recurrence. It is important to distinguish between ctDNA’s prognostic and predictive applications. Prognostic applications are well-established at identifying patients at higher risk of recurrence or death regardless of treatment. Predictive applications, which identify patients who may differentially benefit from specific therapies, remain under investigation. Currently, there are two ctDNA-based strategies for determining MRD: tumor-informed and tumor-agnostic (plasma-only) approaches (Figure 1) [3]. The tumor-informed (TI) approach involves the identification of somatic mutations in tumor tissue. This method allows for the detection of specific genetic alterations that can be used as biomarkers for MRD [9]. On the other hand, the tumor-agnostic (TA) approach relies on the detection of somatic alterations directly from cfDNA using broad-panel sequencing without prior knowledge of tumor-specific mutations [9,10]. Both approaches have shown superior specificity and sensitivity compared with conventional biomarkers such as carcinoembryonic antigen (CEA) in multiple studies [11,12,13]. As a result, ctDNA-based monitoring shows significant promise for refining MRD detection, improving recurrence risk stratification, and supporting risk-adapted adjuvant treatment strategies. Beyond early-stage CRC, emerging evidence and ongoing clinical trials continue to explore the role of ctDNA in monitoring the treatment response, detecting molecular resistance, and guiding therapeutic decisions in metastatic CRC [10,14]. This review summarizes current evidence for the prognostic and predictive utility of ctDNA in CRC, emphasizing its role in adjuvant and metastatic disease in light of the current literature.

## 2. Technical Approaches of ctDNA Detection: Tumor-Informed vs. Tumor-Agnostic

Despite the superiority of ctDNA in the detection of microscopic disease when compared to conventional biomarkers such as CEA, substantial differences exist between the two primary ctDNA-monitoring approaches: tumor-informed and tumor-agnostic [15]. The tumor-informed strategy primarily utilizes whole-exome sequencing or targeted next-generation sequencing of patient tissue to identify tumor-specific mutations, which are then tracked in plasma samples. Comparatively, the tumor-agnostic approach uses predefined gene panels or methylation markers to identify tumor-driven alterations in cfDNA without prior tumor genomic characterization. These approaches differ significantly in their analytical performance, particularly in the MRD setting, where ctDNA is present at extremely low levels. Recent bivariate meta-regression analysis of 27 studies revealed that in the landmark setting (1 month following completion of definitive therapy), there was no statistically significant difference in sensitivity between the two approaches (TI vs. TA: 48% vs. 58%, *p* = 0.070) [16]. Subsequently, when ctDNA was tracked serially, the tumor-informed approach demonstrated a substantially superior sensitivity compared to the tumor-agnostic assay (TI vs. TA: 88% vs. 59%, *p* = 0.001). Both approaches boasted high specificity in the landmark (TI vs. TA: 92% vs. 90%, *p* = 0.329) and serial settings (TI vs. TA: 91% vs. 88%, *p* = 0.419) [16]. Importantly, accuracy varies substantially across commercial and academic assays in both tumor-agnostic and tumor-informed methods of ctDNA detection. This was evident in the bivariate analysis performed by Camblor et al., which showed high heterogeneity, especially pronounced in the serial setting (I^2^ = 86.4%; *p* < 0.0001) compared to the landmark setting (I^2^ = 69.0%; *p* < 0.0001) [16]. Comparisons between the two modalities are summarized in Table 1 below.

### 2.1. Tumor-Agnostic Approach

The tumor-agnostic approach uses plasma-only analysis using predefined gene panels to identify ctDNA without requiring patient-specific tissue. This methodology offers several advantages, including a faster turnaround time, broader applicability when tissue is unavailable or insufficient, and detection of a wider range of mutations. However, a key limitation to this option is a higher detection threshold. This approach typically requires variant allele frequencies (VAFs) ≥ 0.1%, compared to ≥0.01% in tumor-informed assays. Importantly, 80% of mutations detected during recurrence surveillance fall below the 0.1% VAF detection threshold, limiting sensitivity in the MRD setting [17]. Additionally, tumor-agnostic methods carry an increased risk of false positives from clonal hematopoiesis mutations, as these approaches lack the ability to distinguish tumor-derived from age-related somatic alterations. Parikh et al. analyzed ctDNA samples from 70 patients with stage I-IV CRC using a tumor-agnostic assay, demonstrating at the landmark time point a recurrence sensitivity of 55.6% and specificity of 100%. Incorporation of serial sampling and surveillance sampling (within 4 months of recurrence) improved sensitivity to 69% and 91%, respectively. Integrating genomics and epigenomic signals, including aberrant methylation patterns, provided an average 30% increase in sensitivity compared to either modality alone [11].

### 2.2. Tumor-Informed Approach

In contrast, the tumor-informed approach utilizes whole-exome sequencing or targeted next-generation sequencing of patient tumor tissue to create personalized panels that target patient-specific mutations for MRD detection in plasma. This system provides superior sensitivity, with detection thresholds of ≥0.01% VAF [17], improving MRD detection beyond that of tumor-agnostic approaches. In a direct head-to-head comparison, tumor-informed methods detected ctDNA in 66% of stage I–III patients pre-operatively compared to only 31% with the tumor-agnostic assay (*p* = 0.008). Both assays had detection rates of 100% in the stage IV population [17]. This performance gap in early-stage disease may be explained by the tumor-informed assays’ ability to detect mutations at significantly lower VAFs, demonstrating a median surveillance VAF of 0.028% (range 0.018–0.783%), well below the ≥0.1% threshold of the tumor-agnostic assay [17]. However, these tumor-informed approaches require adequate tumor tissue and longer turnaround times compared to a tumor-agnostic approach, which may make this option infeasible in certain patients.

## 3. ctDNA as a Prognostic Biomarker in Colorectal Cancer

### Minimal Residual Disease Detection

MRD refers to a microscopic tumor burden that persists 2–10 weeks after curative-intent surgery yet remains undetectable by conventional imaging modalities. The emergence of ctDNA detection techniques has led to significant advances in the detection of MRD in CRC, providing a minimally invasive approach to capturing microscopic levels of disease that would otherwise be undetectable during clinical and radiological assessments. Recent studies have demonstrated the role of ctDNA in prognosticating the recurrence risk at the individual level, identifying which patients are most likely to benefit from post-surgical adjuvant chemotherapy, and reducing treatment-related toxicity by avoiding overtreatment [7,12,18].

The prognostic utility of post-operative ctDNA testing as a marker indicating the risk of recurrence or death regardless of treatment was established in 2016 through a prospective analysis of patients with stage II colon cancer conducted by Tie et al. [19]. In this study, post-operative ctDNA was assessed in plasma from 230 patients with resected stage II colon cancer to identify residual disease. Patients who were MRD-positive after surgery and did not receive adjuvant chemotherapy (ACT) exhibited a markedly increased risk of radiologic recurrence compared with the MRD-negative patients (HR 18, 95% CI 7.9–40), with a 3-year RFS of 0%. In contrast, ctDNA-negative patients had comparatively favorable oncologic outcomes, with a 3-year RFS of 90%, comparable to stage I CRC, which is conventionally managed with surgery alone. In this study, following stratification of patients into clinicopathological low-risk and high-risk groups, the post-operative ctDNA status improved RFS risk estimates for both low-risk (HR 28, 95% CI 8.1–93) and high-risk (HR 7.5, 95% CI 2.6–22) groups, supporting the prognostic value of ctDNA independent of clinicopathological features.

Furthermore, in this same study, ctDNA positivity at the completion of ACT was associated with a substantially increased risk of subsequent radiologic recurrence (HR 11, 95% CI 1.8–68), indicating a worse prognosis in patients with a ctDNA-positive status following treatment. During the study’s surveillance period, serial ctDNA measurements appeared more sensitive compared to carcinoembryonic antigen (CEA) measurements. Moreover, 85% of patients who developed radiologic recurrence had detectable ctDNA at or before the time of imaging-detected relapse, compared to 41% that demonstrated elevated CEA levels.

This prognostic significance of post-operative ctDNA was corroborated and expanded by the GALAXY trial (observational arm of the CIRCULATE-Japan study), which is one of the largest prospective cohorts evaluating ctDNA-based MRD detection in CRC. This observational-arm study analyzed the pre-surgical and post-surgical ctDNA in 2240 patients with stage II-IV colorectal cancer, with a median follow-up of 23 months [20]. The 4-week post-surgical ctDNA positivity was associated with a higher recurrence risk, an inferior disease-free survival (DFS), and an inferior overall survival (OS). Among 336 out of 2109 patients with ctDNA positivity in the MRD window (2–10 weeks following surgery), 78.27% (263/336) experienced recurrence, whereas only 13.14% (223/1773) of ctDNA-negative patients in this same timeframe experienced recurrence (HR 11.99, 95% CI 10.02–14.35, *p* < 0.0001). This translated to a 24-month DFS of 20.57% versus 85.10% in ctDNA-positive and ctDNA-negative patients, respectively. An extended follow-up further highlighted this difference in ctDNA-positive versus ctDNA-negative patients, exhibiting a 36-month DFS of 16.7% (95% CI 12.1–21.9%) versus 83.5% (95% CI 81.2–85.6%), respectively. Importantly, following multivariate analysis for DFS, post-surgical ctDNA positivity emerged as the most significant prognostic factor for poor DFS (HR 12.08, 95% CI 9.56–15.27, *p* < 0.001) and OS (HR 9.87, 95% CI 5.60–17.40, *p* < 0.001) in patients with stage II–III CRC in comparison with other conventional clinicopathological risk factors. An overview of completed clinical trials assessing the prognostic and predictive utility of ctDNA-based MRD detection in CRC is provided in Table 2.

## 4. ctDNA as a Predictive Biomarker in Colorectal Cancer

### Treatment Response Prediction

While ctDNA’s prognostic value in identifying high-risk patients is established, its emerging role as a predictive biomarker in identifying which patients may derive a benefit from ACT may greatly extend the therapeutic benefits of ctDNA. Recent studies suggest that ctDNA may be used to determine which patient populations are more likely to benefit from chemotherapy.

The GALAXY study provided robust evidence that patients with a post-surgical positive ctDNA status may derive a significant benefit from ACT compared to ctDNA-negative patients. In the initial analysis from 2023, ctDNA-positive patients with stage II or stage III CRC assessed at 4 weeks after surgery demonstrated markedly improved DFS when treated with ACT compared with those who did not receive adjuvant therapy (adjusted HR 6.59, 95% CI 3.53–12.3, *p* < 0.0001) [20]. This effect was observed across all pathological stages when analyzed separately, with adjusted HRs of 5.84 (95% CI 1.36–25.1, *p* = 0.018) for high-risk stage II disease, 7.02 (95% CI 3.46–14.2, *p* < 0.0001) in stage III, and 4.0 (95% CI 1.85–8.8, *p* < 0.0001) in stage IV CRC. Notably, three out of four ctDNA-positive patients with stage I or low-risk stage II CRC who did not receive ACT experienced recurrence, suggesting a potential benefit of therapy in lower-risk populations when MRD is present. In contrast, among the 531 high-risk stage II or stage III patients who were ctDNA-negative at 4 weeks after surgery, no statistically significant benefit of adjuvant chemotherapy was observed when comparing patients who received ACT with those who did not (adjusted HR 1.71, 95% CI 0.80–3.7; *p* = 0.167). This finding occurred despite 41.2% of patients receiving treatment. At 18 months, disease-free survival rates were notably similar between groups: 94.9% for the ACT-treated group (95% CI 91.0–97.2%) and 91.5% for the observation groups (95% CI 87.6–94.2) [18].

The latest publication of the GALAXY study from 2024 published newer analyses with an extended cohort size (2240 patients) and follow-up period (23 months). These analyses confirmed a benefit from ACT treatment in ctDNA-positive patients (adjusted HR 0.23, 95% CI 0.15–0.35, *p* < 0.0001), while no benefit from chemotherapy was seen in the ctDNA-negative patient population (adjusted HR 0.70, 95% CI 0.46–1.06, *p* = 0.091). These updated findings reinforced those from the original 2023 publication, further indicating the ctDNA status as a potential predictive biomarker in the treatment response within the CRC patient population.

Complementary evidence for the utility of ctDNA regarding its predictive value for treatment was provided by an analysis of ctDNA data from the phase III IDEA-France trial [25], as well as providing information concerning the optimal duration of treatment. This 2019 study analyzed ctDNA data from the larger IDEA-France trial [26], where 2010 eligible patients with stage III CRC were treated with either 3 or 6 months of chemotherapy, with a median follow-up of 4.3 years. Of these 2010 patients, 805 had available ctDNA samples prior to the initiation of chemotherapy and had ctDNA values during and after treatment. This study found a superior 2-year DFS in the 6-month treatment arm compared to the 3-month treatment arm for both the ctDNA-negative group (HR 0.69, 95% CI 0.52–0.93, *p* = 0.015) as well as the ctDNA-positive group (HR 0.50, 95% CI 0.27–0.95, *p* = 0.033) [27]. In this study, the benefit seen from extended treatment appeared more pronounced in the ctDNA-positive group in comparison to the ctDNA-negative group, suggesting that patients with detectable residual disease may require prolonged therapy to achieve optimal disease control.

An important study to note is the phase II/III COBRA study, which sought to identify if low-risk stage II colon cancer patients with ctDNA positivity after resection would benefit from adjuvant chemotherapy. In this trial, patients without traditional high-risk features were randomized post-operatively in a 1:1 fashion to two arms, with a primary endpoint of ctDNA clearance at 6 months following surgery. In Arm A, patients were treated with standard-of-care observation, while in Arm B, patients were managed with ctDNA-directed therapy (6 months of adjuvant doublet chemotherapy in this study). This randomized trial was terminated early due to a higher incidence of ctDNA clearance within the observation arm. During the 6-month monitoring period, investigators did not observe any improvement in ctDNA clearance using a ctDNA-directed treatment strategy in this low-risk population, showing a 43% clearance rate in the observation arm (95% CI 10–82%) compared to 11% (95% CI 0.3–48%) in the chemotherapy arm (*p* = 0.98). Therefore, the study was stopped for futility. The findings highlight the limitations of ctDNA-guided strategies in low-risk stage II colon cancer and underscore the need for refined risk stratification. Specifically, better methods beyond conventional anatomic staging are needed to identify patient subgroups that may derive a clinical benefit from ctDNA-based biomarkers [28]. Several ongoing large-scale randomized clinical trials designed to evaluate ctDNA-guided treatment strategies are summarized in Table 3.

## 5. ctDNA-Guided Adjuvant Treatment Management

Considering accumulating evidence and ongoing studies, ctDNA-guided treatment approaches support the role of ctDNA in adjuvant treatment management by enabling the detection of MRD and facilitating risk-adapted, individualized treatment decision-making following curative-intent surgery [12,18].

In 2022, Tie et al. expanded upon the use of ctDNA in the DYNAMIC trial, which evaluated ctDNA-guided decision-making regarding adjuvant chemotherapy in patients with stage II CRC (Table 2) [8]. In this randomized trial, 455 patients were assigned in a 2:1 ratio to have treatment decisions guided by ctDNA results or conventional clinicopathological features. In the ctDNA-guided arm, post-operative ctDNA-positive patients (tested at weeks 4 and 7) received ACT, while ctDNA-negative patients were observed. In the standard management arm, decisions regarding ACT use were guided by conventional clinicopathological criteria. When evaluating the primary endpoint of 2-year RFS, this study observed that ACT use was significantly reduced by the ctDNA-guided approach (15% ctDNA-guided vs. 27.9% standard, RR 1.82, 95% CI 1.25 to 2.65). The reduction in chemotherapy utilization did not compromise the 2-year RFS between the ctDNA-guided arm and the standard arm (93.5% vs. 92.4%, respectively, absolute difference 1.1%, 95% CI −4.1 to 6.2). Similarly, 3-year RFS was comparable (91.7% and 92.4%, respectively; HR 0.96, 95% CI 0.51 to 1.82), supporting the non-inferiority of the ctDNA-guided approach while limiting ACT use.

Updated outcomes of this study were published in 2025, with confirmation of the reduction in ACT use seen in the ctDNA-guided approach without compromising long-term outcomes [24]. Upon re-assessment at an extended follow-up, the 5-year RFS remained virtually identical between treatment arms: 88.3% with ctDNA-guided therapy compared to 87.2% for standard care (HR 0.96, 95% CI 0.61 to 1.53, *p* = 0.858). Overall survival at this extended follow-up was similarly non-inferior between the treatment arms, with 5-year OS rates of 93.8% for the ctDNA-guided arm and 93.3% for the standard arm (HR 1.05, 95% CI 0.47 to 2.37, *p* = 0.887). Disease-specific survival was 97.9% in the ctDNA-guided arm versus 97.2% in the standard arm (HR 1.19, 95% CI 0.35 to 4.09, *p* = 0.79). This further illustrates that MRD-directed treatment decisions based on ctDNA do not adversely affect long-term cancer-specific outcomes in stage II CRC.

Beyond a binary application to ctDNA-guided treatment approaches, recent evidence has provided insights into the potential significance of an approach to therapy guidance using the quantitative ctDNA burden, defined as the number of tumor-derived mutations per milliliter of plasma. A 2020 pooled analysis of three separate cohort studies by Tie et al. elucidated an increased recurrence risk with an increasing ctDNA mutant allele frequency (MAF), with hazard ratios of 1.2, 2.5 and 5.8 for MAFs of 0.1%, 0.5% and 1%, respectively [29]. Within this patient population, the 3-year RFS was markedly worse in patients with ctDNA above the median MAF of 0.046% (9% without ACT, 25% with ACT), compared to patients below the median MAF (33% without ACT, 70% with ACT). This highlights the potential of ctDNA quantification for prognosticating long-term outcomes.

Within the DYNAMIC trial, stratification of the post-operative ctDNA burden demonstrated significant prognostic value with treatment guidance. In patients treated with ACT following curative-intent surgery, those with a higher-than-median post-operative ctDNA burden had inferior ctDNA clearance rates (75%) following ACT treatment compared to those with a lower-than-median post-operative ctDNA burden (100%) (*p* = 0.047) [30]. Along with this, high-ctDNA-burden patients had worse 5-year RFS subsequently in comparison to low-burden patients (58.9% high burden versus 95.2% low burden, HR 10.62, *p* = 0.005). Collectively, these findings demonstrate that increasing ctDNA assessment extends beyond a binary MRD classification. The quantitative ctDNA burden provides clinically meaningful prognostic information that may further refine risk stratification and inform future treatment personalization strategies in stage II CRC.

Standard practice for the treatment of stage III CRC consists of curative-intent surgical resection followed by ACT. However, despite the routine use of ACT, data indicate that more than 50% of patients with stage III disease are effectively cured by surgery alone, raising concerns regarding potential overtreatment in this population [31]. The BESPOKE study, a multi-center, prospective observational study, sought to investigate the utility of using ctDNA for guidance of ACT treatment decisions in stage II and stage III CRC patients [32]. Out of 1792 patients initially enrolled in this cohort, outcomes for 1001 patients with stage II/III CRC were analyzed. Longitudinal ctDNA testing was prospectively performed, collected during the MRD window (2–12 weeks post-operatively) and surveillance window (post-ACT or 12 weeks post-operative for observed patients). Following curative resection, 62.4% (625/1001) of patients received ACT: 25.9% of stage II patients and 91.3% of stage III patients. In the stage III patient population with available ctDNA results during the MRD window, ctDNA positivity was observed in 24.9% of patients (126/505). A positive ctDNA status during the MRD window in stage III disease was associated with inferior DFS (HR 10.1, *p* < 0.0001). In this study, 33.3% of stage III patients in the observation cohort tested ctDNA-positive during the surveillance window and were associated with inferior DFS (HR 34.1, *p* = 0.0008) compared to those testing ctDNA-negative. In the ACT cohort, 21.1% of stage III patients tested ctDNA-positive, also presenting with a poor DFS (HR 54.6, *p* = 0.0001) compared to the corresponding ctDNA-negative population. In MRD-negative patients with stage II–III disease, the 18/24-month DFS rates were 93.0% and 91.7%, respectively, whereas in the MRD-positive group, these rates were 44.4% and 41.4%.

To further investigate the use of ctDNA-guided therapy in stage III colon cancer patients, analysis from the DYNAMIC-III trial has recently been published, investigating ctDNA-informed management versus standard care with regard to escalation and de-escalation of care [24].

In the DYNAMIC-III trial, 1002 eligible patients with stage III resected colon cancer underwent 1:1 randomization to either ctDNA-informed or standard-of-care management. ctDNA testing was performed 5–6 weeks after surgery. Within the ctDNA-informed cohort, ACT was escalated to FOLFOXIRI (for 3 months, extended to 6 months in patients who tolerated treatment) following a positive ctDNA result and de-escalated following a negative ctDNA result. De-escalation options included a shortened chemotherapy duration, reduced regimen intensity (most commonly oxaliplatin-based doublet regimen to single-agent fluoropyrimidine), or observation. Of the 968 evaluable patients, 482 were randomized to the ctDNA-informed arm (353 ctDNA-negative, 129 ctDNA-positive) and 479 patients to the standard management arm (349 ctDNA-negative, 130 ctDNA-positive).

In the ctDNA-negative cohort, the ctDNA-guided treatment de-escalation strategy did not meet the prespecified non-inferiority margin for DFS compared to the standard management arm (85.3% versus 88.1%, respectively). Therefore, non-inferiority, defined as maintaining recurrence-free survival within a prespecified acceptable margin compared with standard therapy, was not formally demonstrated for ctDNA-guided treatment de-escalation in the ctDNA-negative population. Nevertheless, this finding indicates that chemotherapy de-escalation can reduce toxicity and treatment exposure. However, a small potential reduction in efficacy cannot be excluded, highlighting the importance of individualized risk–benefit discussions. Among ctDNA-negative patients with clinically low-risk disease (T1-3N1), the point estimate was consistent with the pre-set noninferiority margin (3-year RFS 91.0% ctDNA-guided vs. 93.2% standard arm); however, this subgroup finding was not statistically significant. Interestingly, ctDNA-guided management did markedly reduce the rates of treatment-related hospitalizations (8.5% ctDNA-guided vs. 13.2% standard arm, relative risk (RR) = 0.64, 95% CI 0.42–1.00, *p* = 0.047). It also reduced rates of high-grade acute adverse events of special interest (6.2% vs. 10.6%, RR = 0.59, 95% CI 0.35–0.98, *p* = 0.037) when compared to standard care.

Within the ctDNA-positive patient cohort, ACT escalation did not demonstrate an improvement in survival based on the ctDNA-guided approach compared to the standard management approach (2-year RFS 51% vs. 61%, respectively; HR 1.16, 95% CI 0.87–1.53). Therefore, while the DYNAMIC-III trial reinforced the prognostic significance of post-operative ctDNA detection, ctDNA-guided treatment escalation or de-escalation in stage III colon cancer did not result in outcomes comparable to those achieved with standard adjuvant chemotherapy. This highlights the necessity for further studies exploring how ctDNA can be effectively integrated with therapeutic strategies to improve patient outcomes.

### 5.1. ctDNA Clearance as Indicator of Treatment Response

Beyond the baseline ctDNA status, ctDNA clearance following treatment and ctDNA dynamics during treatment have emerged as a prospective intermediate endpoint that may serve as an early indicator of treatment response, with the potential to guide adaptive treatment strategies.

The GALAXY study demonstrated that at 24 weeks post-surgery, 68.48% (63/92) of patients receiving ACT achieved ctDNA clearance, compared to just 12.2% (11/90) of the observation arm (adjusted HR 8.50, 95% CI 4.2–17.3, *p* < 0.0001) [20]. Among patients with ctDNA positivity who received ACT, markedly inferior DFS was demonstrated in patients who did not achieve ctDNA clearance (adjusted HR 11, 95% CI 5.2–23.0, *p* < 0.0001). This finding suggests that ctDNA clearance may help identify a subset of patients who remain resistant to standard ACT.

The updated 2024 GALAXY analysis refined this assessment by distinguishing between sustained clearance, transient clearance, and no clearance of ctDNA in a cohort of 181 ctDNA-positive patients [20]. Patients achieving sustained clearance (37.56%) demonstrated dramatically better outcomes, with a DFS of 89.0% and OS of 100% at 24 months, and with an event rate (defined as rate of disease recurrence or death) of 10.29%. In contrast, those with transient clearance (32.04%) experienced markedly worse outcomes, with an event rate of 86.21% (DFS HR 19.72, *p* < 0.0001; OS HR 25.51, *p* = 0.0007), while those who never achieved clearance (30.39%) fared the worst, with an event rate of 100% (DFS HR 124.76, *p* < 0.0001; OS HR 75.62, *p* < 0.0001). The stark differences in outcomes between these clearance patterns underscore the value that continued MRD clearance can provide in terms of disease-free survival.

Findings from the randomized DYNAMIC trial in stage II colon demonstrated that a ctDNA-guided adjuvant treatment strategy reduced chemotherapy use without compromising long-term recurrence-free or overall survival, while also highlighting the prognostic relevance of the post-operative ctDNA burden and ctDNA clearance following treatment [30].

In 2022, Henriksen and colleagues reported results from a prospective observational study of 168 patients with stage III CRC who were treated with curative-intent surgery followed by serial ctDNA monitoring, seeking to explore the potential benefit of serial analysis of ctDNA in this patient population [13]. The researchers qualified patients received standard ACT following surgery per physician discretion. Patients were followed by serial ctDNA monitoring at multiple time points: post-operatively, during ACT, after ACT (within 3 months of therapy completion), and throughout routine follow-up (every 3 months). The primary endpoint was set as recurrence-free survival. The status of post-operative ctDNA positivity was noted to be a strong predictor of recurrence (HR 7.0, 95% CI 3.7–13.5, *p* < 0.001). Notably, a positive ctDNA result directly following ACT treatment was an even stronger predictor of recurrence (HR 50.76, 95% CI 15.4–167, *p* < 0.001). This pronounced hazard ratio for post-treatment ctDNA positivity further highlights the evidence that persistent residual disease despite standard ACT can identify a patient population at a high risk of recurrence.

### 5.2. ctDNA During Surveillance of Colorectal Cancers

Furthermore, Henriksen et al. interestingly found that serial 3-month ctDNA analysis provided a median 9.8-month lead time for detecting recurrence compared to standard computed tomography imaging [13]. Corroborating this is the 2021 prospective, observational study by Chen and colleagues, in which 240 patients with stage II/III CRC underwent curative-intent surgery followed by serial ctDNA monitoring [33]. In this study, following surgery, ACT was administered at the physician’s discretion per the standard of care. Patients underwent monitoring of ctDNA at multiple time points: pre-operatively, post-operatively (day 3–7), at 6 months after surgery, and serially every 3 months until month 24. The primary outcome was recurrence-free survival. This study demonstrated that post-operative ctDNA-positivity (8.3% of patients) was strongly associated with the recurrence risk (HR 10.98, 95% CI 5.31–22.72, *p* < 0.001), with a recurrence rate of 60% in ctDNA-positive patients, compared to 11% in ctDNA-negative patients. Among patients included in surveillance, ctDNA positivity was associated with a high recurrence risk (HR 32.02, 95% CI 10.79–95.08, *p* < 0.001). Importantly, among the 23 patients who experienced recurrence following surgery, 19 patients (82.6%) had serial ctDNA positivity. This serial monitoring provided a mean lead time of 5.01 months (*p* = 0.002) in identifying recurrence of disease over computed tomography, with an overall accuracy of 92.0%. These increased lead times provide a rationale for ctDNA monitoring following curative-intent treatment to allow for potentially earlier therapeutic intervention. However, whether this earlier molecular detection can markedly improve outcomes remains unproven. Further investigational studies are warranted to determine whether the advanced lead time over radiologic recurrence translates to improved patient outcomes.

### 5.3. ctDNA in Metastatic Colorectal Cancer

ctDNA analysis can provide a non-invasive method for identifying molecular alterations that predict a response or resistance to targeted therapy in metastatic colorectal cancer (mCRC) [34].

In the phase III PARADIGM trial, baseline ctDNA analysis was used to explore the presence of resistance-associated genomic alterations, including mutations in KRAS, NRAS, PTEN, and the EGFR extracellular domain, as well as HER2 and MET amplifications and ALK, RET, and NTRK1 fusions, prior to the initiation of first-line therapy. Patients with detectable alterations were classified as ctDNA-positive. A “hyperselected” subgroup was subsequently defined as patients with RAS wild-type, left-sided tumors in whom none of these resistance-associated alterations were detected. In this subgroup, treatment with panitumumab plus mFOLFOX6 was associated with numerically longer overall survival compared with bevacizumab plus mFOLFOX6 (median OS: 40.7 vs. 34.4 months; HR, 0.76). In contrast, the presence of ctDNA-detected alterations appeared to attenuate the potential benefit of anti-EGFR-based therapy. Broadly comparable survival outcomes were observed between treatment groups, irrespective of primary tumor sidedness. Collectively, these observations suggest that ctDNA-based molecular profiling may represent a promising approach to refining patient selection for anti-EGFR therapies in mCRC [35].

**Figure 2:**
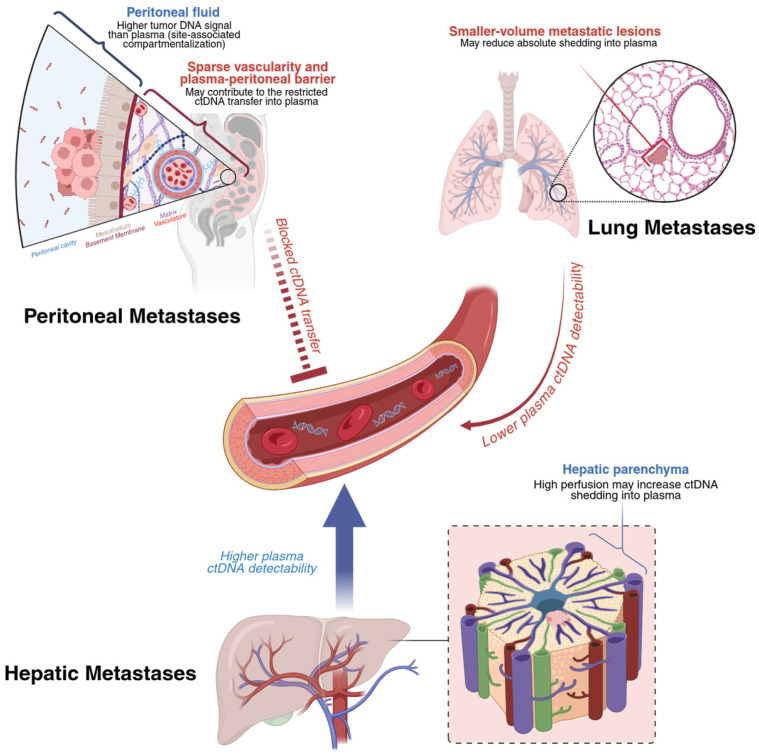
Proposed contributors to low plasma ctDNA shedding by metastatic site in colorectal cancer. Illustration created with BioRender.com.

Urbini et al. investigated whether ctDNA in liquid biopsy could reliably detect druggable gene mutations compared with tumor tissue analysis and assess its potential to predict the prognosis and monitor patients with mCRC receiving first-line bevacizumab-based chemotherapy. Using a Next-Generation Sequencing (NGS) panel, they identified a strong concordance between ctDNA liquid biopsy and tumor tissue. More specifically, out of 48 patients with KRAS, NRAS, and BRAF mutations, the concordance levels were significant at 100% (48 of 48), 97.9% (47 of 48), and 97.9% (47 of 48), respectively. In this study, low levels of ctDNA in the serum had longer progression-free survival (PFS; 15.9 vs. 12.2 months, *p* = 0.02). Moreover, patients who had a substantial drop in initial ctDNA levels showed improved PFS (12.3 vs. 8.2 months, *p* = 0.04) and OS rates (34.1 vs. 11.1 months, *p* = 0.03) compared to those who did not experience this decline. They also compared ctDNA levels with the tumor size, measured by contrast-enhanced computed tomography (CECT), at different time points to evaluate whether a correlation existed between the two. The results demonstrated that decreases in ctDNA levels were accompanied by reductions in tumor size, while increases in ctDNA levels were associated with tumor growth. Notably, increases in ctDNA levels were observed before the radiological onset of disease progression. These results highlight ctDNA’s potential as a minimally invasive biomarker in detecting the tumor burden, monitoring the response to therapy, and predicting progression earlier compared to standard imaging techniques. However, ctDNA levels may be unreliable in patients with isolated peritoneal metastases who have a low tumor cell burden, reducing the sensitivity of this biomarker [36]. In patients with CRC, plasma cfDNA levels are substantially higher in patients with liver metastases than in patients with peritoneal metastases, highlighting site-specific differences in ctDNA shedding. In one study, plasma cfDNA was detectable in only 20% of patients with CRC peritoneal metastases compared with 93% of patients with liver metastases (*p* < 0.0001) [36]. Notably, the same study reported that tumor-derived cfDNA is enriched within the peritoneal compartment. The mutant allele fraction in peritoneal fluid was markedly higher than in plasma (median 16.4% vs. 0.28%, *p* = 0.0019), supporting a compartmentalization model where peritoneal implants shed cfDNA locally instead of into systemic circulation [36]. In a large Danish cohort of 851 stage II–III CRC patients, post-operative ctDNA detection among patients who eventually recurred was particularly poor for those who developed peritoneal metastases. Only 2 out of 10 (20%) of these peritoneal metastases were detectable via ctDNA, representing a significantly elevated false-negative rate. Van’t Erve et al. proposed that low levels of cfDNA with peritoneal metastases may be caused by intrinsic differences in the dissemination pathway of peritoneal metastases, namely that tumor DNA may remained compartmentalized within the peritoneal cavity [36]. This, along with the presence of a “peritoneum–plasma barrier” and the relatively poor vascularization typical of peritoneal implants, may lead to restriction of cfDNA from entering the systemic circulation. These site-specific biological limitations translate to high false-negative rates, which may pose significant clinical challenges, as ctDNA-negative results cannot reliably exclude peritoneal or lung disease. To address this limitation in clinical practice and ongoing studies, plasma ctDNA is interpreted alongside cross-sectional imaging and compartment-directed sampling (ascites or peritoneal lavage) when there is suspected or known peritoneal disease (Figure 2). Peritoneal fluid cfDNA and ctDNA analyses can be used to improve molecular detection and profiling. Van’t Erve et al. reported a cfDNA sensitivity of 100% in peritoneal fluid, and a prospective study by Yuan et al. reported a similarly high diagnostic performance for peritoneal cfDNA, with sensitivity 100% and specificity 77.3% in the training cohort, and sensitivity 83.3% and specificity 100% in a small validation cohort [36,37]. Similarly, lung metastases were detected at a comparably low rate of 19% (4/21 detected) [38]. This low ctDNA detection rate is consistent with observations suggesting that smaller lung lesions may shed insufficient amounts of tumor DNA to reach assay detection thresholds [39]. In contrast, the relatively higher detection rate of ctDNA in liver metastases may reflect a greater metastatic tumor burden and increased proliferative activity, leading to enhanced cellular turnover and augmented release of tumor DNA into the circulation [40]. Additional studies with larger sample sizes must be conducted to achieve higher concordance between ctDNA levels and the tumor tissue mutation status, to be applied in clinical practice [34].

Recent evidence suggests that early changes in ctDNA can serve as a valuable biomarker for predicting treatment efficacy in mCRC. Grancher et al. analyzed 152 first-line mCRC patients from two prospective cohorts, stratifying them by treatment intensity (≤2 drugs vs. ≥3 drugs) and assessing ctDNA at baseline and at the third or fourth chemotherapy cycle. Pretreatment ctDNA was detectable in 84.9% of patients. A substantial decrease in ctDNA levels (ΔctDNA ≥ 80% and undetectable) was more frequently observed in patients receiving more intensive regimens (51.5% vs. 32.7%, *p* = 0.015). This early ctDNA clearance was associated with an improved treatment response and significantly longer PFS and OS, with the median OS reaching 30.2 months in the intensive treatment group compared with 16.4 months in patients with minimal ctDNA reduction. Importantly, a ≥80% reduction in ctDNA from baseline accompanied by complete ctDNA clearance at cycle 3–4 (ΔctDNA ≥ 80%_undetectable) remained an independent predictor of OS after multivariate analysis. These findings indicate that early ctDNA clearance reflects the treatment intensity and serves as a strong prognostic biomarker in first-line mCRC, supporting its potential role in guiding therapy [41].

### 5.4. ctDNA as a Biomarker in Colorectal Cancer with Resectable Liver Metastases

Liver metastasis represents one of the most common metastatic patterns in CRC, with approximately 25–30% of patients developing hepatic metastases during the course of their disease [42]. The presence of liver metastases has a profound impact on disease staging, therapeutic decision-making, and long-term survival outcomes. In recent years, growing interest in non-invasive biomarkers capable of reflecting the tumor burden and real-time disease dynamics has led to increasing focus on circulating tumor-derived DNA-based approaches. These approaches have emerged as a promising tool for monitoring the treatment response, detecting minimal residual disease, and stratifying the recurrence risk, particularly in patients with colorectal cancer liver metastases [43].

Liu et al. investigated ctDNA’s predictive value for the tumor response and risk of recurrence after liver resection for colorectal liver metastasis (CRLM). They tracked ctDNA levels before and after pre-operative chemotherapy in patients with colorectal liver metastasis undergoing liver resection. Their results ultimately showed that patients who had detectable ctDNA before chemotherapy but became undetectable afterward experienced substantially longer recurrence-free survival (median: 17 months vs. 7 months) and hepatic recurrence-free survival (unreached vs. 8 months) compared to those whose ctDNA remained detectable. Moreover, ctDNA clearance after chemotherapy was significantly associated with a major pathologic response in the resected liver metastases (53.4% vs. 32.1%). In addition to ctDNA levels, the association between the tumor regression grade and radiological response to pre-operative chemotherapy was evaluated; however, no significant correlation was observed, suggesting that ctDNA may represent a more reliable biomarker for predicting the tumor response. These findings suggest that ctDNA positivity after chemotherapy can support the predictive value and risk of recurrence in patients with CRC undergoing curative-intent resection for liver metastases. However, a negative ctDNA result, even with a favorable tumor regression grade, does not guarantee freedom from recurrence. When ctDNA results were combined with the pathological tumor regression grade, patients who remained ctDNA-positive after chemotherapy and had a minor pathological response had an 89.5% likelihood of recurrence. In contrast, those who were ctDNA-negative with a major pathological response had a substantially lower recurrence rate of 51.5% [44].

Another distinguishing study by Liu et al. used NGS to compare the CRLM-specific J25 panel (a panel with fewer genes and greater sequencing depth) with a broader 624-gene panel (a panel with a greater number of genes but smaller sequencing depth). It was found that the J25 panel was more accurate and sensitive for predicting recurrence. In this prospective cohort of patients with CRLM after hepatectomy, ctDNA positivity at post-operative day 6 emerged as a significant predictor of DFS. It is also important to note that several factors may play a role in shaping the DFS rate. These multivariable factors include the ctDNA status, post-operative CA19-9 value, bilobar or unilobar localization, metastasis lesion number, pre-operative chemotherapy, pre-operative CA19-9 value, and KRAS mutation. The independent factors that are correlated with the prognosis are ctDNA positivity and no pre-operative chemotherapy. Another important limitation is that ctDNA was assessed at a single post-operative time point rather than through serial measurements across multiple post-operative time points. Additionally, the 624-gene panel was only paired with 25 genes, thereby calling into question the generalizability of the results. However, the results of DFS and predictive sensitivity of the recurrence rate in the J25 panel bring hope to future studies, which may further provide an opportunity to establish ctDNA as a primary biomarker to predict recurrence in patients with CRLM [45].

### 5.5. Clinical Utility of ctDNA in dMMR/MSI-H Metastatic Colorectal Cancer

The use of immune checkpoint inhibitors (ICIs) has significantly improved treatment outcomes in patients with mismatch repair-deficient/microsatellite instability-high (dMMR/MSI-H) mCRC. Despite these advances, primary resistance and disease progression during ICI therapy remain clinically relevant issues [46]. Furthermore, there is ongoing uncertainty regarding the optimal duration of immunotherapy and the timing of treatment discontinuation in patients who achieve a complete response. ctDNA may represent a potentially useful tool for monitoring the treatment response and for early detection of treatment resistance in this patient population [47].

In the SAMCO-PRODIGE 54 trial, 132 patients with dMMR/MSI-H metastatic colorectal cancer were randomized to receive either avelumab or standard chemotherapy with or without targeted therapy. This prespecified secondary analysis evaluated ctDNA dynamics as a potential biomarker, with progression-free survival (PFS) as the primary endpoint. ctDNA was detectable in 83.8% of baseline plasma samples. Baseline ctDNA positivity showed limited prognostic value for the median PFS (4.5 vs. 8.2 months; HR 1.32) and OS (20.0 months vs. not reached; HR 1.79). In contrast, early reduction in ctDNA at one month (median cutoff −86%) was strongly predictive: favorable ctDNA responders had a median PFS of 12.0 months versus 2.4 months for poor responders (HR 2.98; *p* < 0.001) and OS not reached versus 14.0 months (HR 3.61; *p*  < 0.001). The predictive value was particularly pronounced in the avelumab arm (PFS HR 4.22; OS HR 17.40). Combining ctDNA dynamics with radiological assessment further improved long-term survival prediction. These results indicate that early ctDNA changes may serve as a robust non-invasive biomarker to monitor ICI efficacy and guide treatment decisions in dMMR/MSI-H mCRC [48].

Recent evidence suggests that analysis of ctDNA, particularly the assessment of microsatellite instability in plasma (ctDNA-MSI), may represent a promising biomarker in patients with dMMR/MSI-H mCRC treated with ICIs. In this prospective, single-center study, 54 patients, most of whom had metastatic disease (78%), were longitudinally monitored before and during therapy using droplet digital PCR to measure ctDNA-MSI and cfDNA. The study demonstrated that higher baseline ctDNA-MSI levels were associated with poorer PFS and OS. On-treatment ctDNA-MSI kinetics strongly predicted the response to ICI therapy and outperformed cfDNA kinetics. Patients with decreasing or undetectable ctDNA-MSI exhibited a 24-month PFS rate of 77%, whereas those with increasing ctDNA-MSI had a 24-month PFS rate of 0% (HR 7.93; 95% CI, 2.23–28.21; *p* = 0.005). Moreover, OS was 11.3 months in patients with rising ctDNA-MSI, while the median OS was not reached in patients with decreasing or negative ctDNA-MSI (*p* < 0.0001), indicating substantially improved outcomes in these groups. Collectively, these findings support ctDNA-MSI as a feasible and clinically relevant biomarker that reflects tumor dynamics. Its use may help guide treatment decisions in dMMR/MSI colorectal cancer, enabling a more sensitive and timely evaluation of the therapeutic response compared with conventional cfDNA measurements [49].

### 5.6. Limitations of ctDNA-Based Assessment of MRD in Colorectal Cancer

Although ctDNA shows a strong prognostic performance across several CRC studies, there are several important limitations that may affect interpretations and clinical generalizability within the current body of literature. First, ctDNA may not be reliably shed into plasma across all disease patterns. Post-operative plasma ctDNA sensitivity is notably poor for peritoneal or lung metastases, which may produce clinically significant false-negative results [36,37,38]. Mechanistically, compartmentalization and reduced vascularity in the peritoneal cavity appear to contribute to this limitation, as patients with isolated colorectal peritoneal metastases have markedly higher cfDNA levels in peritoneal fluid compared with plasma [36]. Therefore, a negative plasma ctDNA result should be interpreted with caution in clinical contexts where low plasma ctDNA shedding occurs. This limitation remains a key barrier to the use of plasma ctDNA as a standalone surveillance strategy.

Second, there is substantial heterogeneity in assay design and analytical performance, which may explain differing results across study settings. For example, tumor-informed (personalized) assays typically prioritize MRD sensitivity but face the limitations of requiring tumor tissue and longer turnaround times. In contrast, tumor-agnostic approaches may offer wider clinical feasibility without the need for matched tumor tissue, supporting use in tissue-limited settings, allowing more rapid assay deployment, and accommodating heterogeneous or evolving tumor clones. However, this approach could be limited by sensitivity at very low tumor fractions and faces confounding effects from clonal hematopoiesis [3,5,6]. In addition to assay design, pre-analytic variables (sample handling, cfDNA yield, timing of post-operative draws) and interpretive thresholds (clearance metrics) further contribute to variability, and they can affect the sensitivity and specificity of ctDNA-based assays [3].

Third, while substantial evidence points to the prognostic value of ctDNA, its predictive value for adjuvant chemotherapy is unresolved and requires further evidence. In an interventional study conducted by Tie et al., ctDNA-guided management reduced adjuvant chemotherapy use without a significant difference in recurrence-free survival in stage II colon cancer patients. In comparison, the COBRA clinical trial was halted after a prespecified analysis found no improvement in ctDNA clearance with adjuvant chemotherapy compared with observation among ctDNA-positive, low-risk stage II patients [28]. As such, although ctDNA positivity identifies patients at high risk of recurrence, existing interventional data do not consistently demonstrate that ctDNA-positive disease is preferentially responsive to standard chemotherapy.

Additionally, defining clinical utility endpoints remains challenging. Earlier molecular detection of recurrence may introduce lead-time bias. This is particularly relevant if earlier treatment does not result in significant improvements in disease-free survival, overall survival, or patient-centered outcomes (e.g., reduced treatment-related toxicity burden, improved quality of life). In addition, costs, access, variability in reimbursement, and logistical constraints (for example, the need for tumor tissue in tumor-informed approaches) provide further operational barriers to the implementation of ctDNA-based assays as a clinically relevant diagnostic tool [3].

Lastly, economic and logistical considerations present potential barriers to widespread adoption, though investigations into these barriers remain limited. Preliminary cost-effectiveness analysis utilizing data from major ctDNA trials suggested cost savings with the addition of MRD detection by ctDNA. This analysis found that the implementation of ctDNA led to an average cost saving of $9771 and would likely be cost effective in the US healthcare system [50]. A separate budget impact analysis based on ctDNA-guided treatment reduction rates from the DYNAMIC trial indicated cost reduction for both commercial insurance and Medicare Advantage payers [51]. However, these findings are limited by their modeling-based approaches, and by variability in reimbursement.

## 6. Conclusions and Future Directions

In early-stage CRC, the identification of patients who derive a true benefit from ACT continues to rely largely on conventional clinicopathological criteria, despite their limited ability to accurately stratify the recurrence risk and predict the treatment benefit. Accumulating evidence consistently supports the prognostic value of ctDNA in CRC and demonstrates an emerging predictive potential for guiding adjuvant treatment decisions. The randomized DYNAMIC trial demonstrated that ctDNA-guided management in stage II colon cancer safely reduces ACT utilization without compromising disease-free survival. However, outcomes in high-risk (T4) tumors suggested that a single post-operative ctDNA assessment may be insufficient for definitive risk stratification [8]. More recent findings from the DYNAMIC-III trial further confirmed the prognostic value of post-operative ctDNA in stage III disease and showed that ctDNA-guided escalation and de-escalation strategies reduced chemotherapy exposure particularly among clinically low-risk patients without clear evidence of compromised survival. However, formal non-inferiority was not established [24].

Observational studies consistently report a greater benefit from ACT among ctDNA-positive patients, whereas ctDNA-negative patients appear to derive limited benefit. Nevertheless, these findings warrant cautious interpretation due to the predominantly non-interventional design of these studies and the inclusion of heterogeneous stage II–III populations [18,52]. Exploratory analyses from randomized trials, including CALGB/SWOG 80702, further suggest that the ctDNA status may identify subgroups with differential benefits from adjunctive therapies, supporting its potential predictive relevance [53]. Ongoing large-scale randomized trials (Table 3) are expected to provide more definitive evidence regarding the role of ctDNA in guiding treatment escalation or de-escalation strategies in CRC [54].

In mCRC, longitudinal ctDNA dynamics have been associated with the treatment response, progression-free survival, overall survival, and the emergence of resistance mechanisms, as supported by meta-analyses and longitudinal monitoring studies [38,55]. However, important limitations remain, including reduced sensitivity in settings such as isolated peritoneal metastases and a low tumor burden, assay heterogeneity, and lack of prospective interventional validation. These limitations underscore the need for standardized methodologies and further clinical trials before routine implementation [10,38,56,57].

Moving into the future, several key priorities could help advance ctDNA’s clinical integration in CRC diagnostics and management. Completion of ongoing randomized controlled trials (CIRCULATE-Japan, CIRCULATE-North America) will more definitively establish whether ctDNA-guided treatment strategies improve patient outcomes and which clinical decision points are most likely to bring a benefit. Addressing technical limitations—particularly the low sensitivity in peritoneal and lung metastases—through compartment-specific sampling and multimodal biomarker approaches may improve detection accuracy in low-shedding disease. Furthermore, optimizing tumor-agnostic approaches will improve sensitivity in patient populations where tumor-informed assays are suboptimal options. Combining ctDNA with orthogonal biomarkers and imaging may further mitigate false negatives and refine risk stratification. Critically, investigating whether early intervention based on MRD detection improves outcomes compared to radiologic surveillance will address lead-time bias concerns and establish clinical endpoints of ctDNA utilization. Finally, though investigations of the cost-effectiveness of these assays are emerging, research is still quite limited. Therefore, cost-effective analyses and implementation studies are critically needed to support reimbursement decisions and equitable access in these patient populations.

In summary, ctDNA represents a highly promising, non-invasive biomarker in CRC that provides real-time insights into the tumor burden, treatment response, and emerging resistance mechanisms. While its prognostic value is well-established, the predictive role of ctDNA in guiding treatment personalization continues to evolve. With ongoing prospective validation and methodological standardization, ctDNA is poised to become an integral component of precision oncology by meaningfully improving individualized therapeutic decision-making processes across the CRC disease continuum.

## Figures and Tables

**Table 1:** Comparison of tumor-informed and tumor-agnostic ctDNA detection approaches for MRD assessment.

Characteristic	Tumor-Informed (Personalized)	Tumor-Agnostic (Plasma-Only)
Tumor Tissue Requirement	Required	Not required
Turnaround Time	4–6 weeks	7–14 days
Detection Threshold (VAF)	≥0.01%	≥0.1%
Analytical Performance		
Pre-operative Detection (Stages I–III)	66% [17]	31% [17]
Sensitivity—Landmark (1-Month Post-Therapy)	48% [16]	58% [16] (NS)
Sensitivity—Serial Monitoring	88% [16]	59% [16]
Specificity—Landmark	92% [16]	90% [16] (NS)
Specificity—Serial Monitoring	91% [16]	88% [16] (NS)
Clinical Considerations		
Clonal Hematopoiesis Interference	Lower risk of false positives	Higher risk of false positives
Primary Advantages	Superior MRD sensitivity Superior in serial monitoring Lower false-positive rate	Rapid results Feasible when tissue is unavailable Captures tumor heterogeneity
Primary Limitations	Requires adequate tumor tissue Longer turnaround time	Reduced sensitivity at low VAF Inferior detection threshold

Abbreviations: VAF, variant allele frequency; MRD, minimal residual disease; NS, not significant. Note: Performance values represent estimates from meta-analysis. Performance varies between assays (heterogeneity I^2^ = 69–86.4%) [16].

**Table 2:** Select list of completed trials for prognostic and predictive value of ctDNA.

Author, Year	Staging	Number of Patients	ctDNA Technique	Timing of ctDNA Assessment	ctDNA Positivity Rate	ctDNA Negativity Rate	Recurrence Rate for ctDNA-Positive	Recurrence Rate for ctDNA-Negative
Tie et al., 2016 [19]	Stage II colon cancer	230	Safe-SeqS	4–10 weeks post-op	14/178 (7.9%) post-operative 14/178 (7.9%) (not treated) 6/52 (11%) (with ACT)	164 of 178 (92.1%) post-op	11/14 (78.6%) (no chemo)	16/164 (9.8%) (no chemo)
Schøler et al., 2017 [21]	Stage I-IV CRC	45	ddPCR	Day 0 pre-op Days 8 and 30 post-op Every 3 months up to 3 years	74% (20/27) pre-op 100% (14/14) in relapsing patients post-op 28.6% (6/21) within 3-months post-op	0% (0/12) in non-relapsing patients post-op 71.4% (15/21) within 3-months post-op	100% (6/6)	26.7% (4/15)
Reinert et al., 2019 [22]	Stage I–III CRC	125	Natera Multiplex PCR-based NGS	Pre-op, day 30 post-op, every third month up to 3 years	108/122 (88.5%) overall 10/94 (10.6%) 30 days post-op 7/58 (12.1%) post-ACT 15/75 (20%) longitudinal	84/94 (89.4%) 30 days post-op 51/58 (87.9%) post-ACT 60/75 (80%) longitudinal	7/10 (70%) 30 days post-op 7/7 (100%) post-ACT 14/15 (93.3%) longitudinal	10/84 (11.9%) 30 days post-op 7/51 (13.7%) post-ACT 2/60 (3.3%) longitudinal
Tarazona et al., 2019 [23]	Stage I–III colon cancer	94	Targeted NGS + ddPCR	At baseline 6–8 weeks post-op Every 4 months for up to 5 years	60/94 (63.8%) baseline 14/69 (20.3%) 6–8 weeks post-op	55/69 (79.7%) 6–8 weeks post-op	8/14 (57.1%)	13%
Parikh et al., 2021 [11]	Stage I–IV CRC	84	Tumor-agnostic Guardant Reveal	4 weeks post-op 4 weeks after ACT completion	17/70 (24%) at end of definitive therapy	49/70 (70%) at end of definitive therapy and with >1 year of follow-up	15/17 (88%), but 15/15 (100%) patients with >1 year follow-up recurred	12/49 (24.5%)
Tie et al., 2022 [7]	Stage II colon cancer	441	Safe-SeqS	Weeks 4 and 7 post-op	45/291 (15.5%) in ctDNA-guided group	246/291 (84.5%) in ctDNA-guided group	8/45 (18%)	15/246 (6%)
Henriksen et al., 2022 [13]	Stage III CRC	168	Signatera multiplex-PCR NGS Assay	At diagnosis 2–8 weeks post-op Serially through ACT treatment Post-ACT, every 3-month surveillance period	20/140 (14%) post-op 10/93 (11%) post-ACT	120/140 (86%) post-op 83/93 (89%) post-ACT	16/20 (80%) post-op 9/10 (90%) post-ACT	22/120 (18%) post-op 8/83 (10%) post-ACT
Nakamura et al., 2024 [20]	Stage II–III colon cancer; Stage IV CRC	2109	Signatera PCR-NGS assay	2–10 weeks post-op and before ACT Surveillance samples at 4, 12, 24, 36, 48, 72 and 96 weeks until recurrence	336/2109 (15.93%) in MRD window 310/1791 (17.3%) positive at any point in surveillance	1773/2109 (84.07%) in MRD window 1481/1791 (82.7%) serially negative	263/336 (78.27%) 261/310 (84.19%) in surveillance window	233/1773 (13.14%) 89/1481 (6.01%) in surveillance window
Tie et al., 2025 [24]	Stage II colon cancer	441	SaferSeqS Assay	Weeks 4 and 7 post-op	45/291 (15.5%) in ctDNA-guided arm	246/291 (84.5%)	8/45 (17.8%)	16/246 (6.5%)

**Table 3:** Select list of ongoing trials for prognostic and predictive value of ctDNA.

Trial Name NCT Number	Population	Patient N	Primary Endpoints	Brief Summary
CIRCULATE-US NCT05174169	Stage III colon cancer	1912	ctDNA positive status 5-year DFS	ctDNA-guided adjuvant therapy selection
BESPOKE CRC NCT04264702	Stage I-IV CRC	1788	2-year recurrence rate	Impact of ctDNA on adjuvant treatment decisions
ALTAIR phase III NCT04457297	Stage III colon or rectal cancer w/post-operative ctDNA positivity	240	3-year DFS	Preemptive therapy in ctDNA-positive patients
ERASE-CRC NCT05062889	Stage III and high-risk stage II CRC	477	ctDNA clearance post-ACT	Escalation strategies to increase ctDNA clearance
TRACC NCT04050345	Stage I–III CRC	1000	3-year DFS	Serial ctDNA monitoring and ACT de-escalation
SU2C ACT3 clinical trial NCT03803553	Stage III–IV colon cancer	400	5-year DFS Clearance rate of ctDNA	Additional therapy in ctDNA-positive mCRC
cmPAT NCT06167967	Stage III CRC	990	3-year RFS	ctDNA methylation-guided ACT escalation/de-escalation
AGITG Dynamic-III ACTRN12617001566325	Post-resection Stage III colon cancer	961	2- and 3-year RFS	ctDNA-guided ACT escalation/de-escalation

Abbreviations: ACT, adjuvant chemotherapy; DFS, disease-free survival; RFS, recurrence-free survival; mCRC, metastatic colorectal cancer.

## Data Availability

The data underlying this study are extracted from publicly available studies.
